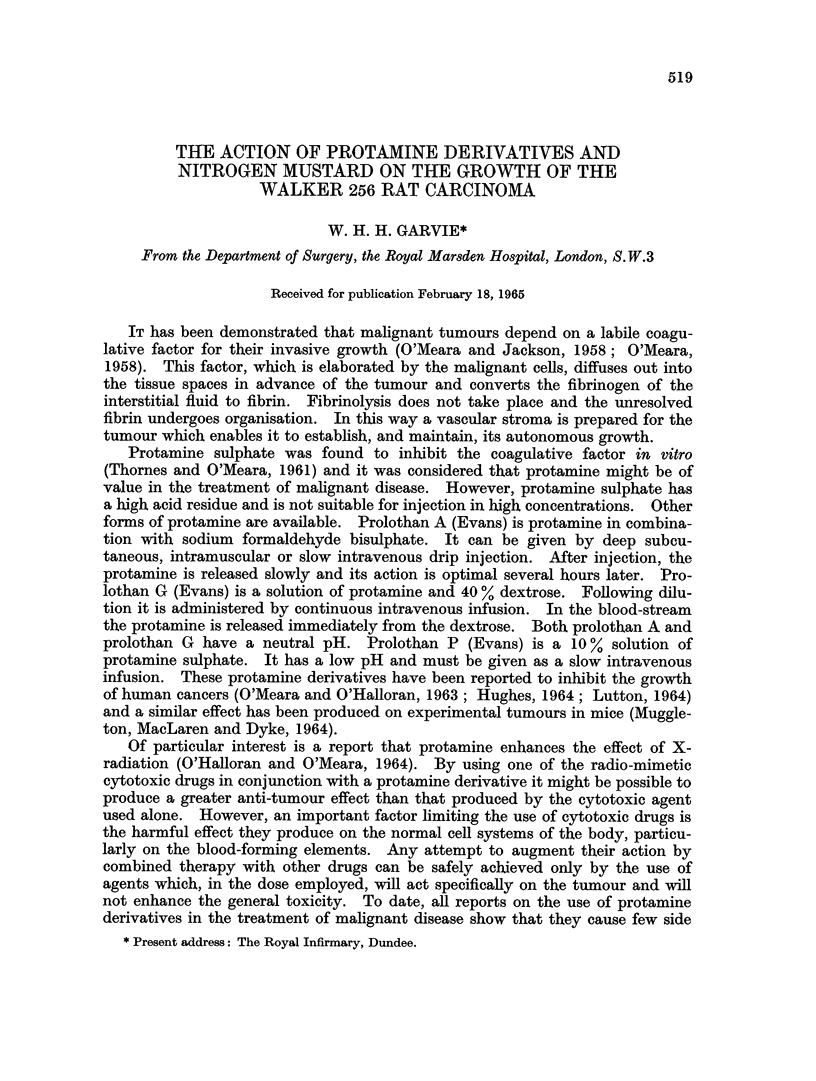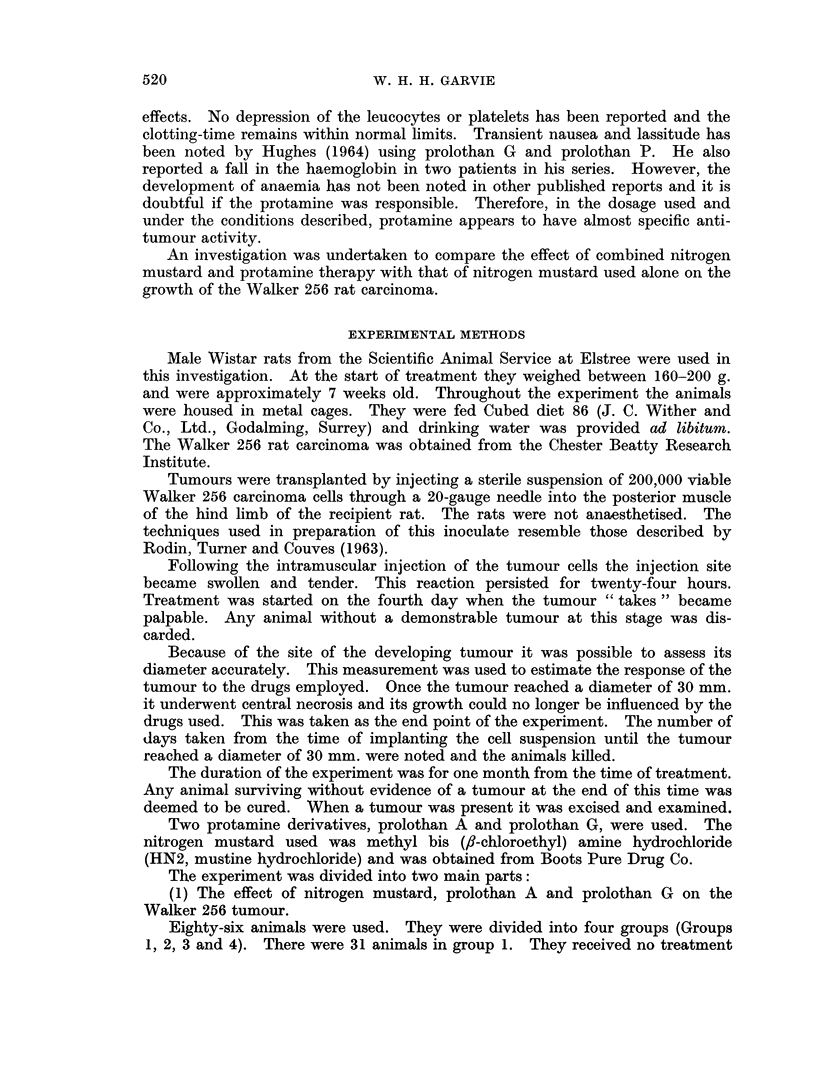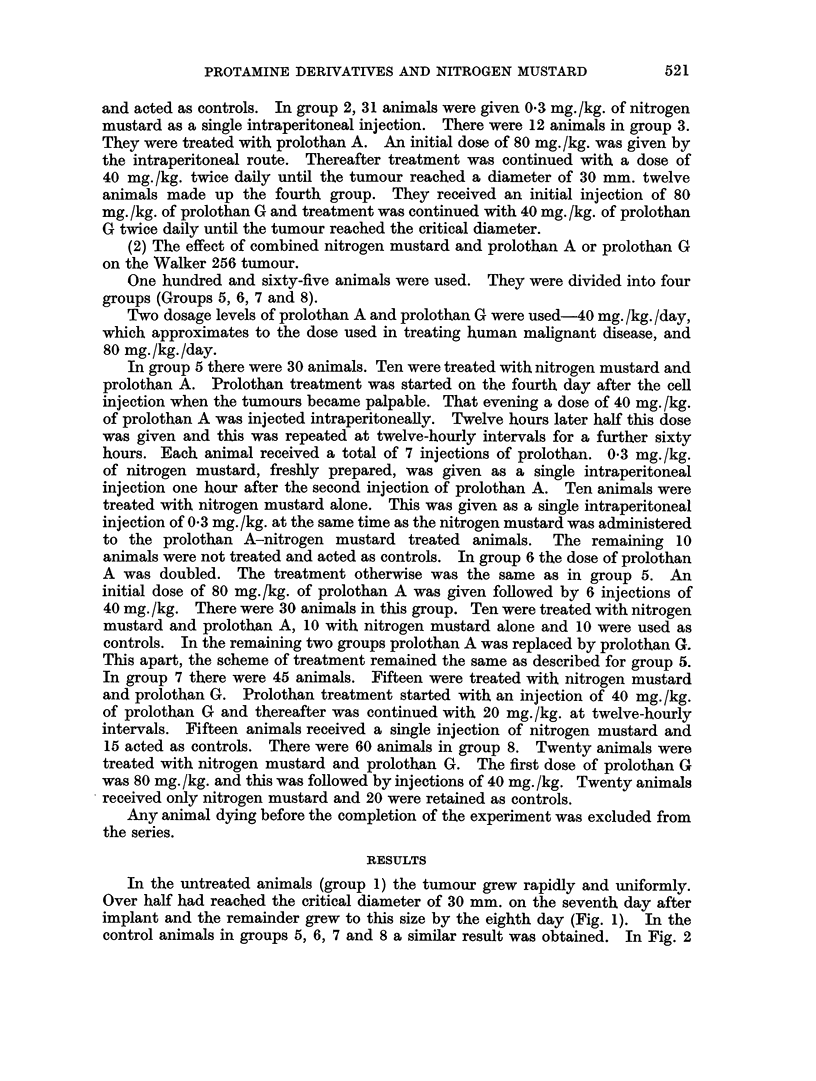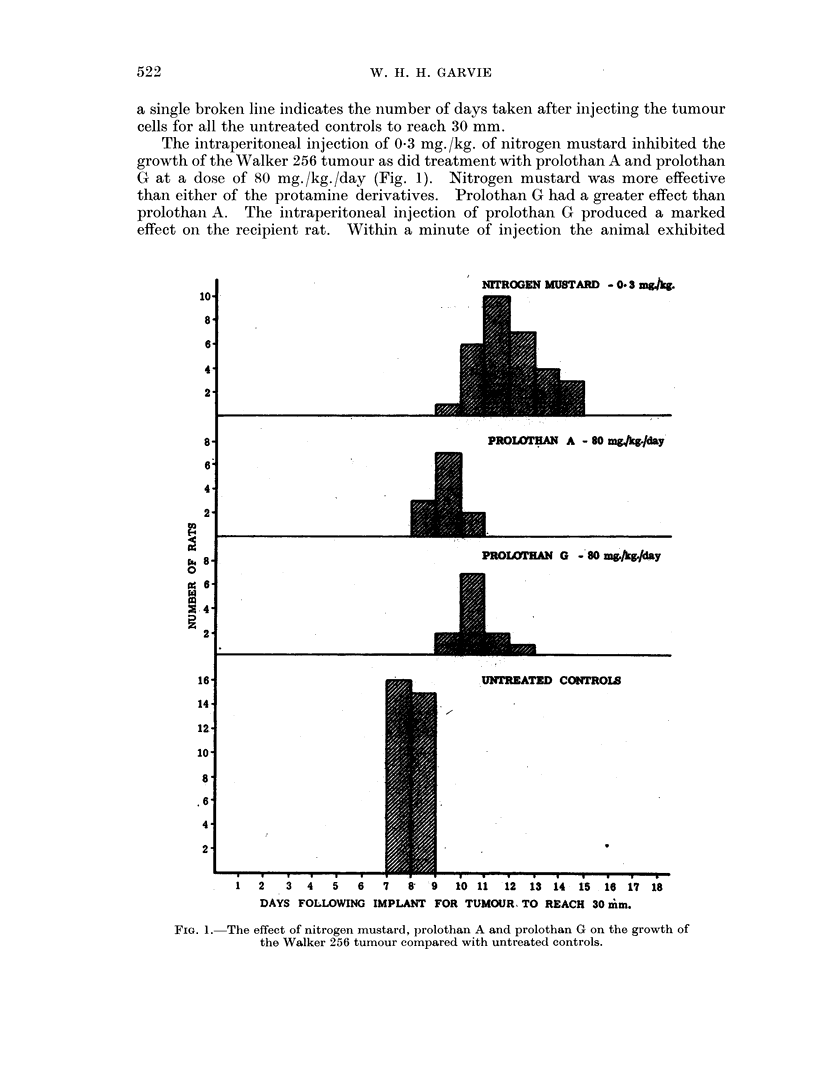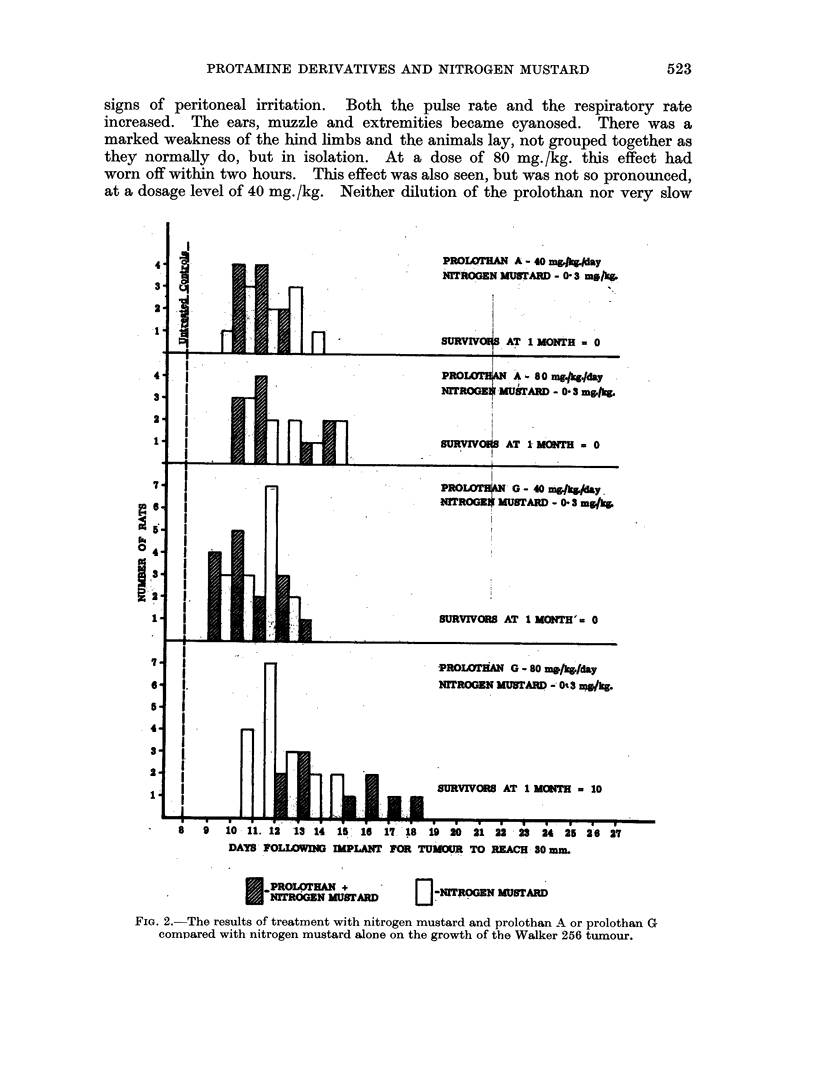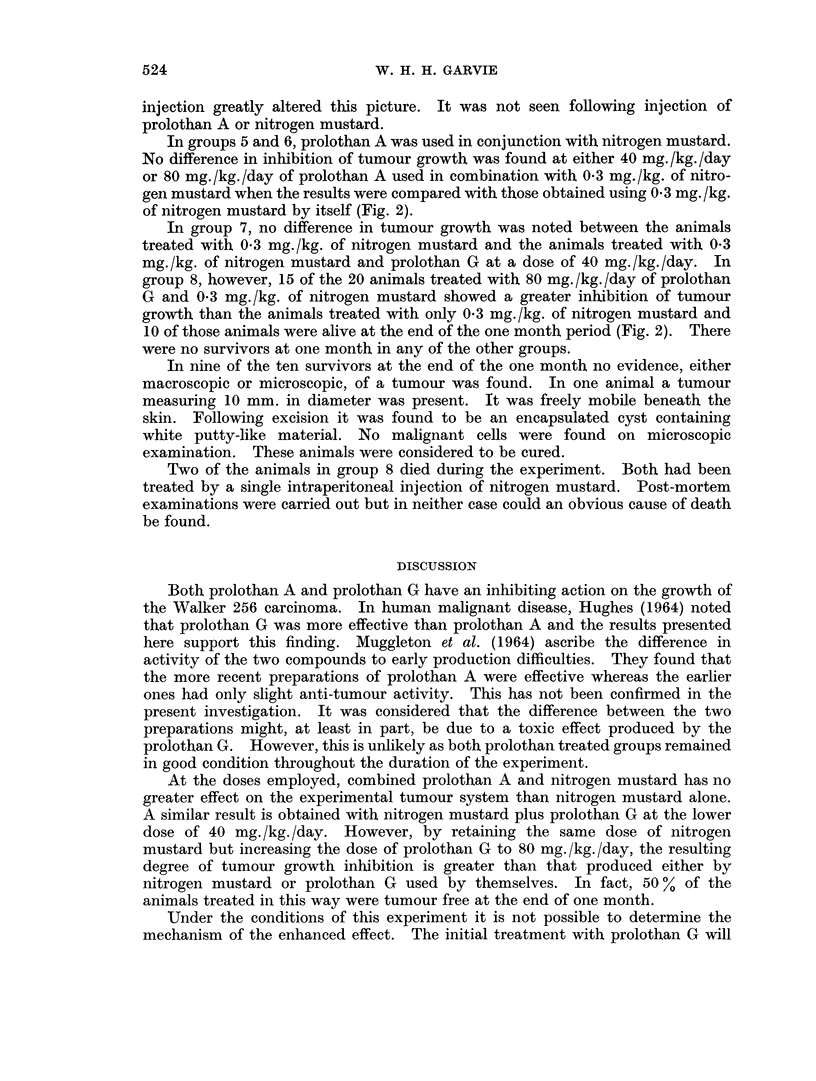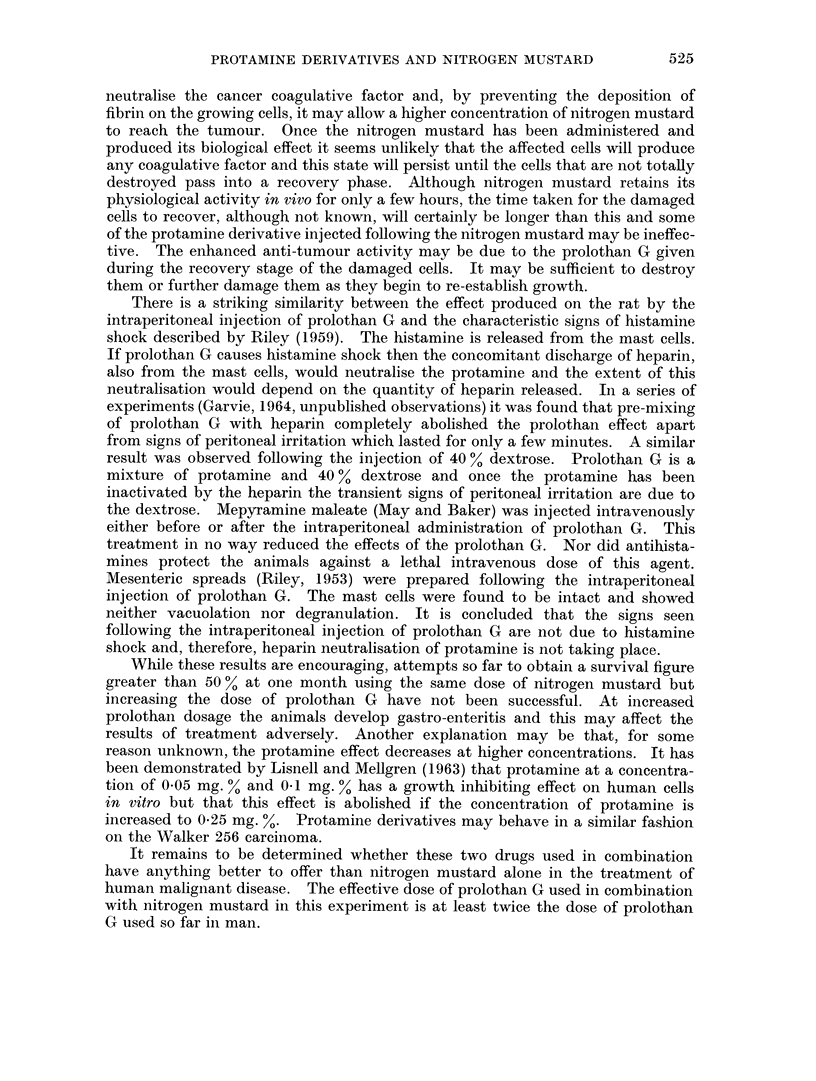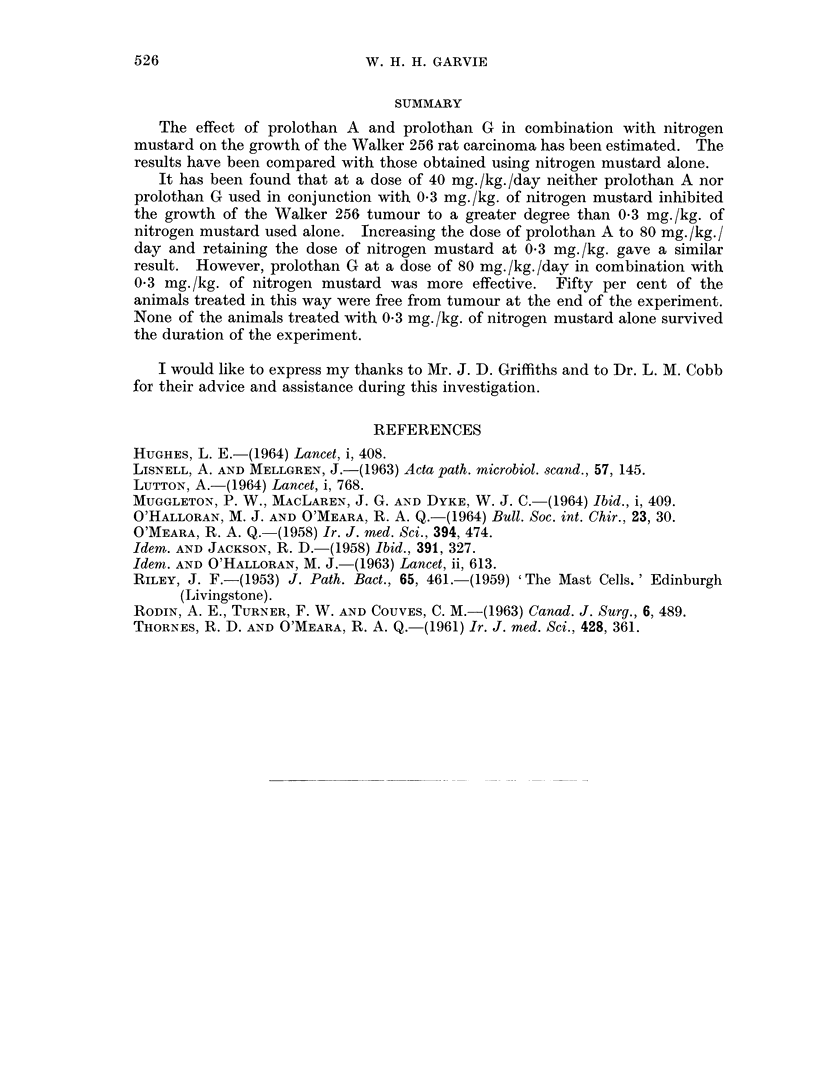# The action of protamine derivatives and nitrogen mustard on the growth of the Walker 256 rat carcinoma.

**DOI:** 10.1038/bjc.1965.63

**Published:** 1965-09

**Authors:** W. H. Garvie


### THE ACTION OF PROTAMINE DERIVATIVES AND NITROGEN MUSTARD ON THE GROWTH OF THE WALKER 256 RAT CARCINOMA

W. H. H. GARVIE*

*From the Department of Surgery, the Royal Marsden Hospital, London, S.W.3*

Received for publication February 18, 1965

IT has been demonstrated that malignant tumours depend on a labile coagulative factor for their invasive growth (O'Meara and Jackson, 1958; O'Meara, 1958). This factor, which is elaborated by the malignant cells, diffuses out into the tissue spaces in advance of the tumour and converts the fibrinogen of the interstitial fluid to fibrin. Fibrinolysis does not take place and the unresolved fibrin undergoes organisation. In this way a vascular stroma is prepared for the tumour which enables it to establish, and maintain, its autonomous growth.

Protamine sulphate was found to inhibit the coagulative factor *in vitro* (Thornes and O'Meara, 1961) and it was considered that protamine might be of value in the treatment of malignant disease. However, protamine sulphate has a high acid residue and is not suitable for injection in high concentrations. Other forms of protamine are available. Prolothan A (Evans) is protamine in combination with sodium formaldehyde bisulphate. It can be given by deep subcutaneous, intramuscular or slow intravenous drip injection. After injection, the protamine is released slowly and its action is optimal several hours later. Prolothan G (Evans) is a solution of protamine and 40% dextrose. Following dilution it is administered by continuous intravenous infusion. In the blood-stream the protamine is released immediately from the dextrose. Both prolothan A and prolothan G have a neutral pH. Prolothan P (Evans) is a 10% solution of protamine sulphate. It has a low pH and must be given as a slow intravenous infusion. These protamine derivatives have been reported to inhibit the growth of human cancers (O'Meara and O'Halloran, 1963; Hughes, 1964; Lutton, 1964) and a similar effect has been produced on experimental tumours in mice (Muggleton, MacLaren and Dyke, 1964).

Of particular interest is a report that protamine enhances the effect of X-radiation (O'Halloran and O'Meara, 1964). By using one of the radio-mimetic cytotoxic drugs in conjunction with a protamine derivative it might be possible to produce a greater anti-tumour effect than that produced by the cytotoxic agent used alone. However, an important factor limiting the use of cytotoxic drugs is the harmful effect they produce on the normal cell systems of the body, particularly on the blood-forming elements. Any attempt to augment their action by combined therapy with other drugs can be safely achieved only by the use of agents which, in the dose employed, will act specifically on the tumour and will not enhance the general toxicity. To date, all reports on the use of protamine derivatives in the treatment of malignant disease show that they cause few side

* Present address: The Royal Infirmary, Dundee.

effects. No depression of the leucocytes or platelets has been reported and the clotting-time remains within normal limits. Transient nausea and lassitude has been noted by Hughes (1964) using prolothan G and prolothan P. He also reported a fall in the haemoglobin in two patients in his series. However, the development of anaemia has not been noted in other published reports and it is doubtful if the protamine was responsible. Therefore, in the dosage used and under the conditions described, protamine appears to have almost specific anti-tumour activity.

An investigation was undertaken to compare the effect of combined nitrogen mustard and protamine therapy with that of nitrogen mustard used alone on the growth of the Walker 256 rat carcinoma.

### EXPERIMENTAL METHODS

Male Wistar rats from the Scientific Animal Service at Elstree were used in this investigation. At the start of treatment they weighed between 160–200 g. and were approximately 7 weeks old. Throughout the experiment the animals were housed in metal cages. They were fed Cubed diet 86 (J. C. Wither and Co., Ltd., Godalming, Surrey) and drinking water was provided *ad libitum.* The Walker 256 rat carcinoma was obtained from the Chester Beatty Research Institute.

Tumours were transplanted by injecting a sterile suspension of 200,000 viable Walker 256 carcinoma cells through a 20-gauge needle into the posterior muscle of the hind limb of the recipient rat. The rats were not anaesthetised. The techniques used in preparation of this inoculate resemble those described by Rodin, Turner and Couves (1963).

Following the intramuscular injection of the tumour cells the injection site became swollen and tender. This reaction persisted for twenty-four hours. Treatment was started on the fourth day when the tumour " takes " became palpable. Any animal without a demonstrable tumour at this stage was discarded.

Because of the site of the developing tumour it was possible to assess its diameter accurately. This measurement was used to estimate the response of the tumour to the drugs employed. Once the tumour reached a diameter of 30 mm. it underwent central necrosis and its growth could no longer be influenced by the drugs used. This was taken as the end point of the experiment. The number of days taken from the time of implanting the cell suspension until the tumour reached a diameter of 30 mm. were noted and the animals killed.

The duration of the experiment was for one month from the time of treatment. Any animal surviving without evidence of a tumour at the end of this time was deemed to be cured. When a tumour was present it was excised and examined.

Two protamine derivatives, prolothan A and prolothan G, were used. The nitrogen mustard used was methyl bis ($\beta$-chloroethyl) amine hydrochloride (HN2, mustine hydrochloride) and was obtained from Boots Pure Drug Co.

The experiment was divided into two main parts:

(1) The effect of nitrogen mustard, prolothan A and prolothan G on the Walker 256 tumour.

Eighty-six animals were used. They were divided into four groups (Groups 1, 2, 3 and 4). There were 31 animals in group 1. They received no treatment and acted as controls. In group 2, 31 animals were given 0·3 mg./kg. of nitrogen mustard as a single intraperitoneal injection. There were 12 animals in group 3. They were treated with prolothan A. An initial dose of 80 mg./kg. was given by the intraperitoneal route. Thereafter treatment was continued with a dose of 40 mg./kg. twice daily until the tumour reached a diameter of 30 mm. twelve animals made up the fourth group. They received an initial injection of 80 mg./kg. of prolothan G and treatment was continued with 40 mg./kg. of prolothan G twice daily until the tumour reached the critical diameter.

(2) The effect of combined nitrogen mustard and prolothan A or prolothan G on the Walker 256 tumour.

One hundred and sixty-five animals were used. They were divided into four groups (Groups 5, 6, 7 and 8).

Two dosage levels of prolothan A and prolothan G were used—40 mg./kg./day, which approximates to the dose used in treating human malignant disease, and 80 mg./kg./day.

In group 5 there were 30 animals. Ten were treated with nitrogen mustard and prolothan A. Prolothan treatment was started on the fourth day after the cell injection when the tumours became palpable. That evening a dose of 40 mg./kg. of prolothan A was injected intraperitoneally. Twelve hours later half this dose was given and this was repeated at twelve-hourly intervals for a further sixty hours. Each animal received a total of 7 injections of prolothan. 0·3 mg./kg. of nitrogen mustard, freshly prepared, was given as a single intraperitoneal injection one hour after the second injection of prolothan A. Ten animals were treated with nitrogen mustard alone. This was given as a single intraperitoneal injection of 0·3 mg./kg. at the same time as the nitrogen mustard was administered to the prolothan A–nitrogen mustard treated animals. The remaining 10 animals were not treated and acted as controls. In group 6 the dose of prolothan A was doubled. The treatment otherwise was the same as in group 5. An initial dose of 80 mg./kg. of prolothan A was given followed by 6 injections of 40 mg./kg. There were 30 animals in this group. Ten were treated with nitrogen mustard and prolothan A, 10 with nitrogen mustard alone and 10 were used as controls. In the remaining two groups prolothan A was replaced by prolothan G. This apart, the scheme of treatment remained the same as described for group 5. In group 7 there were 45 animals. Fifteen were treated with nitrogen mustard and prolothan G. Prolothan treatment started with an injection of 40 mg./kg. of prolothan G and thereafter was continued with 20 mg./kg. at twelve-hourly intervals. Fifteen animals received a single injection of nitrogen mustard and 15 acted as controls. There were 60 animals in group 8. Twenty animals were treated with nitrogen mustard and prolothan G. The first dose of prolothan G was 80 mg./kg. and this was followed by injections of 40 mg./kg. Twenty animals received only nitrogen mustard and 20 were retained as controls.

Any animal dying before the completion of the experiment was excluded from the series.

### RESULTS

In the untreated animals (group 1) the tumour grew rapidly and uniformly. Over half had reached the critical diameter of 30 mm. on the seventh day after implant and the remainder grew to this size by the eighth day (Fig. 1). In the control animals in groups 5, 6, 7 and 8 a similar result was obtained. In Fig. 2

a single broken line indicates the number of days taken after injecting the tumour cells for all the untreated controls to reach 30 mm.

The intraperitoneal injection of 0·3 mg./kg. of nitrogen mustard inhibited the growth of the Walker 256 tumour as did treatment with prolothan A and prolothan G at a dose of 80 mg./kg./day (Fig. 1). Nitrogen mustard was more effective than either of the protamine derivatives. Prolothan G had a greater effect than prolothan A. The intraperitoneal injection of prolothan G produced a marked effect on the recipient rat. Within a minute of injection the animal exhibited

FIG. 1.—The effect of nitrogen mustard, prolothan A and prolothan G on the growth of the Walker 256 tumour compared with untreated controls.

signs of peritoneal irritation. Both the pulse rate and the respiratory rate increased. The ears, muzzle and extremities became cyanosed. There was a marked weakness of the hind limbs and the animals lay, not grouped together as they normally do, but in isolation. At a dose of 80 mg./kg. this effect had worn off within two hours. This effect was also seen, but was not so pronounced, at a dosage level of 40 mg./kg. Neither dilution of the prolothan nor very slow

FIG. 2.—The results of treatment with nitrogen mustard and prolothan A or prolothan G compared with nitrogen mustard alone on the growth of the Walker 256 tumour.

injection greatly altered this picture. It was not seen following injection of prolothan A or nitrogen mustard.

In groups 5 and 6, prolothan A was used in conjunction with nitrogen mustard. No difference in inhibition of tumour growth was found at either 40 mg./kg./day or 80 mg./kg./day of prolothan A used in combination with 0·3 mg./kg. of nitrogen mustard when the results were compared with those obtained using 0·3 mg./kg. of nitrogen mustard by itself (Fig. 2).

In group 7, no difference in tumour growth was noted between the animals treated with 0·3 mg./kg. of nitrogen mustard and the animals treated with 0·3 mg./kg. of nitrogen mustard and prolothan G at a dose of 40 mg./kg./day. In group 8, however, 15 of the 20 animals treated with 80 mg./kg./day of prolothan G and 0·3 mg./kg. of nitrogen mustard showed a greater inhibition of tumour growth than the animals treated with only 0·3 mg./kg. of nitrogen mustard and 10 of those animals were alive at the end of the one month period (Fig. 2). There were no survivors at one month in any of the other groups.

In nine of the ten survivors at the end of the one month no evidence, either macroscopic or microscopic, of a tumour was found. In one animal a tumour measuring 10 mm. in diameter was present. It was freely mobile beneath the skin. Following excision it was found to be an encapsulated cyst containing white putty-like material. No malignant cells were found on microscopic examination. These animals were considered to be cured.

Two of the animals in group 8 died during the experiment. Both had been treated by a single intraperitoneal injection of nitrogen mustard. Post-mortem examinations were carried out but in neither case could an obvious cause of death be found.

### DISCUSSION

Both prolothan A and prolothan G have an inhibiting action on the growth of the Walker 256 carcinoma. In human malignant disease, Hughes (1964) noted that prolothan G was more effective than prolothan A and the results presented here support this finding. Muggleton *et al.* (1964) ascribe the difference in activity of the two compounds to early production difficulties. They found that the more recent preparations of prolothan A were effective whereas the earlier ones had only slight anti-tumour activity. This has not been confirmed in the present investigation. It was considered that the difference between the two preparations might, at least in part, be due to a toxic effect produced by the prolothan G. However, this is unlikely as both prolothan treated groups remained in good condition throughout the duration of the experiment.

At the doses employed, combined prolothan A and nitrogen mustard has no greater effect on the experimental tumour system than nitrogen mustard alone. A similar result is obtained with nitrogen mustard plus prolothan G at the lower dose of 40 mg./kg./day. However, by retaining the same dose of nitrogen mustard but increasing the dose of prolothan G to 80 mg./kg./day, the resulting degree of tumour growth inhibition is greater than that produced either by nitrogen mustard or prolothan G used by themselves. In fact, 50% of the animals treated in this way were tumour free at the end of one month.

Under the conditions of this experiment it is not possible to determine the mechanism of the enhanced effect. The initial treatment with prolothan G will neutralise the cancer coagulative factor and, by preventing the deposition of fibrin on the growing cells, it may allow a higher concentration of nitrogen mustard to reach the tumour. Once the nitrogen mustard has been administered and produced its biological effect it seems unlikely that the affected cells will produce any coagulative factor and this state will persist until the cells that are not totally destroyed pass into a recovery phase. Although nitrogen mustard retains its physiological activity *in vivo* for only a few hours, the time taken for the damaged cells to recover, although not known, will certainly be longer than this and some of the protamine derivative injected following the nitrogen mustard may be ineffective. The enhanced anti-tumour activity may be due to the prolothan G given during the recovery stage of the damaged cells. It may be sufficient to destroy them or further damage them as they begin to re-establish growth.

There is a striking similarity between the effect produced on the rat by the intraperitoneal injection of prolothan G and the characteristic signs of histamine shock described by Riley (1959). The histamine is released from the mast cells. If prolothan G causes histamine shock then the concomitant discharge of heparin, also from the mast cells, would neutralise the protamine and the extent of this neutralisation would depend on the quantity of heparin released. In a series of experiments (Garvie, 1964, unpublished observations) it was found that pre-mixing of prolothan G with heparin completely abolished the prolothan effect apart from signs of peritoneal irritation which lasted for only a few minutes. A similar result was observed following the injection of 40 % dextrose. Prolothan G is a mixture of protamine and 40 % dextrose and once the protamine has been inactivated by the heparin the transient signs of peritoneal irritation are due to the dextrose. Mepyramine maleate (May and Baker) was injected intravenously either before or after the intraperitoneal administration of prolothan G. This treatment in no way reduced the effects of the prolothan G. Nor did antihistamines protect the animals against a lethal intravenous dose of this agent. Mesenteric spreads (Riley, 1953) were prepared following the intraperitoneal injection of prolothan G. The mast cells were found to be intact and showed neither vacuolation nor degranulation. It is concluded that the signs seen following the intraperitoneal injection of prolothan G are not due to histamine shock and, therefore, heparin neutralisation of protamine is not taking place.

While these results are encouraging, attempts so far to obtain a survival figure greater than 50 % at one month using the same dose of nitrogen mustard but increasing the dose of prolothan G have not been successful. At increased prolothan dosage the animals develop gastro-enteritis and this may affect the results of treatment adversely. Another explanation may be that, for some reason unknown, the protamine effect decreases at higher concentrations. It has been demonstrated by Lisnell and Mellgren (1963) that protamine at a concentration of 0·05 mg. % and 0·1 mg. % has a growth inhibiting effect on human cells *in vitro* but that this effect is abolished if the concentration of protamine is increased to 0·25 mg. %. Protamine derivatives may behave in a similar fashion on the Walker 256 carcinoma.

It remains to be determined whether these two drugs used in combination have anything better to offer than nitrogen mustard alone in the treatment of human malignant disease. The effective dose of prolothan G used in combination with nitrogen mustard in this experiment is at least twice the dose of prolothan G used so far in man.

### SUMMARY

The effect of prolothan A and prolothan G in combination with nitrogen mustard on the growth of the Walker 256 rat carcinoma has been estimated. The results have been compared with those obtained using nitrogen mustard alone.

It has been found that at a dose of 40 mg./kg./day neither prolothan A nor prolothan G used in conjunction with 0·3 mg./kg. of nitrogen mustard inhibited the growth of the Walker 256 tumour to a greater degree than 0·3 mg./kg. of nitrogen mustard used alone. Increasing the dose of prolothan A to 80 mg./kg./day and retaining the dose of nitrogen mustard at 0·3 mg./kg. gave a similar result. However, prolothan G at a dose of 80 mg./kg./day in combination with 0·3 mg./kg. of nitrogen mustard was more effective. Fifty per cent of the animals treated in this way were free from tumour at the end of the experiment. None of the animals treated with 0·3 mg./kg. of nitrogen mustard alone survived the duration of the experiment.

I would like to express my thanks to Mr. J. D. Griffiths and to Dr. L. M. Cobb for their advice and assistance during this investigation.

### REFERENCES

HUGHES, L. E.—(1964) *Lancet*, i, 408.
LISNELL, A. AND MELLGREN, J.—(1963) *Acta path. microbiol. scand.*, **57**, 145.
LUTTON, A.—(1964) *Lancet*, i, 768.
MUGGLETON, P. W., MACLAREN, J. G. AND DYKE, W. J. C.—(1964) *Ibid.*, i, 409.
O'HALLORAN, M. J. AND O'MEARA, R. A. Q.—(1964) *Bull. Soc. int. Chir.*, **23**, 30.
O'MEARA, R. A. Q.—(1958) *Ir. J. med. Sci.*, **394**, 474.
*Idem.* AND JACKSON, R. D.—(1958) *Ibid.*, **391**, 327.
*Idem.* AND O'HALLORAN, M. J.—(1963) *Lancet*, ii, 613.
RILEY, J. F.—(1953) *J. Path. Bact.*, **65**, 461.—(1959) 'The Mast Cells.' Edinburgh (Livingstone).
RODIN, A. E., TURNER, F. W. AND COUVES, C. M.—(1963) *Canad. J. Surg.*, **6**, 489.
THORNES, R. D. AND O'MEARA, R. A. Q.—(1961) *Ir. J. med. Sci.*, **428**, 361.